# A Case of Puerperal Convulsions

**Published:** 1840

**Authors:** John M. Bigelow


ART. XII.—*Clinical Reports to the Fairfield Medical Association, Ohio, embracing a case of Puerperal Convulsions, and a case of Tape Worm apparently causing Herpes.* Communicated for publication by M. Z. KREIDER, M. D., Corresponding Secretary of the Society. November, 1839.

I. *A case of Puerperal Convulsions..* By JOHN M. BIGELOW, M. D.

January 23d, 1834, I was called, early in the morning, to see Mrs. W——, aged seventeen years, of a full and plethoric habit, and reported to have been in the seventh month of her first pregnancy. During the preceding fall she had been troubled with ague in its different forms, and, until within the last three or four weeks, had never enjoyed good health. Within this time she has had a ravenous appetite, and neglected her bowels so much as to become costive at times to a considerable degree. I found that she had passed a sleepless night, in consequence of extreme pain in the head.

She rose about daylight this morning, and attempted to go out but was unable to open the door. One of the family observing this, opened it for her, and, from her appearance and manner, believing that something was wrong with her, watched her. In a few minutes he saw her fall from the porch, which was elevated about two feet from the ground. Calling the rest of the family to his assistance, they ran and picked her up in a *fit*. She continued to have convulsive fits, about every half hour, from this time until I arrived—about eight o'clock A. M. I found her pulse beating with great force, about eighty in the minute, and in the intervals of the fits she complained of intolerable pain in the head, uneasiness and pain in the epigastric region, and almost constant nausea, with frequent vomiting of bilious matter. Not unfrequently she would be taken with a fit while in the act of vomiting. Her face was flushed and considerably swollen.

The character of the convulsions was as follows: She would be seized, suddenly and without premonition, with violent alternate contractions and relaxations of the muscles of the whole system—both sides equally alike. The muscles of her face, eyes and mouth, were twitched in every direction with the utmost celerity; her tongue, although not strongly thrust forward, was wounded between the teeth; respiration almost entirely suspended towards the termination of the fit; her face livid, sometimes almost black; froth, tinged with blood, issued from her mouth and nostrils; and the "peculiar sibilating noise," which Dr. Dewees says so strongly characterizes epileptic convulsions, was emitted by her. The pulse, as stated before, in the intervals, and at the commencement of the fit, was full and tense; but during the convulsions it became rapid, small and thready, and her hands bedewed with a cold and clammy sweat. The convulsions gradually declined, leaving the patient comatose, with stertorous breathing for some time. After these symptoms had entirely subsided, she had not the least recollection of what had passed.

I immediately abstracted about twenty ounces of blood from the arm, and gave an effervescing draught. All the preceding symptoms continuing with unabated severity, I applied sinapisms to the pit of the stomach and feet, and at ten o'clock A. M. bled her again to about thirty ounces. The sinapisms not appearing to stimulate the skin sufficiently, I applied a blister to the pit of the stomach, and the mashed root of horse radish to the wrists and ancles. At 12 o'clock, M., the symptoms of gastric irritation having abated, I gave twenty grains calomel. She has not had a fit since 10 o'clock A. M.

Two o'clock P. M.—Fits re-commenced with equal violence. Took twenty ounces blood. The fits now occurred more frequently, leaving her hardly time to recover from the effects of one before another attacked her. At eight o'clock P. M. she took thirty grains jalap, and the blister that was taken from the pit of the stomach was applied between the shoulders. The difference in the condition of the patient this evening, from what it was in the morning, consisted in the absence of gastric distress and nausea, and, in the intervals, of the convulsions, in so deep a coma, that it was impossible to arouse her. This state continued until one o'clock A. M.

24th, 8 o'clock A. M.—Has not had a fit since one o'clock last night. Pulse one hundred, and without the hardness and force that characterized it yesterday. Sleeps a great deal, but her breathing is easy, regular, and without stertor. When awake she appears restless and uneasy, attempting to get out of bed, and does not appear to know any of her attendants, and is unable to talk. Passed urine last night involuntarily. The cathartic, taken yesterday, not having operated, an enema was now administered, which brought away a large quantity of very fœtid excretions. From the restlessness of the patient, I suspected that labor had commenced, and made an examination to escertain the fact, but found that the os tincæ was perfectly closed.

25th.—The rhubarb and jalap, taken last evening, operated well this morning. Patient much better.

26th.—Continues better, with the exception that she cannot sleep. She has also a disposition to be continually talking.

27th.—Since last evening the patient has had paroxysms that resembled hysteria. Besides the wakefulness, when she now attempts to go to sleep, she imagines that she sees dogs, cats, rats, and every kind of vermin, before her. Sometimes they are dancing before her, and again they appear suspended in the air. Her vision appears to be impaired, and every object that she sees is distorted. The strings which hang about the room, and the cracks of the floor and door, appear to be transformed into snakes and worms. The faces of those with whom she was most familiar appear greasy or full of pimples, or in some other way deformed. The house seems turned upside down and in flames, and the chimney falling to pieces. She sometimes screams with all her energy, and begs to know what hideous monster is coming to carry her off, when she only sees her attendants. At times she trembles excessively, even when she feels no apprehensions of danger, or when apparently there is no paroxysm upon her. In the intervals of these *fits* she appears to be cheerful, and even has a disposition to be garrulous. She had not slept for two days and nights, and the moment she closed her eyes, with a disposition to sleep, the paroxysms would be sure to come upon her. I gave forty drops of tinct. opii, and ordered her to take a pill composed of gum asafœt. gr. 1, and gum opii gr. ss, at bed time, and on the following morning to take a tea spoonful of equal parts of tinct. valerian and tinct. asafœt. every four hours, if the fits should continue.

28th.—Slept well last night, and but a slight disposition to the hysterical fits. Much better.

30th.—Taken with labor this morning, and delivered of a fœtus that, to appearance, had been dead about a week.

REMARKS.

There are several points of consideration in this case which render it interesting. Was it a case of "epileptic puerperal convulsions?" or was it "hysterical puerperal convulsions?" From the first, I was led to believe that the brain was in the most imminent danger, and, directing my treatment accordingly, in the course of six hours took upwards of seventy ounces of blood. Had I vaccillated at this time in my treatment, suspecting, as I did, that it *might* be hysteria, and suspended the abstraction of blood, after the loss of sixteen to thirty ounces, as laid down in our best books on this subject, I feel confident that my patient's life would have been forfeited. The supervention of hysteria, at the end of the fourth day, renders the diagnosis of the case doubtful. Although fully aware of the manifold characters assumed by hysteria, yet I do not recollect ever having seen or heard of a case like the one now before us. Indeed, the similarity of the symptoms to those induced by the excessive use of ardent spirits—*mania a potu*—was so highly marked as to excite our observation and astonishment. In her convalescence, which was rapid, she had a preternatural desire for food and drinks of the most highly stimulating character. On the class of disorders commonly denominated *nervous*, there is the greatest variety of opinions, both in regard to their pathology and therapeutics. Indeed, from the investigations of Teale, Macculloch, Mitchell, Tate and others, almost a total revolution of opinion has taken place in regard to these affections, and diseases that once were supposed to be of the most inflammatory character, now, according to their nomenclature, should be classed among the nervous. Prescribing for the name of a disease, we know, has too frequently been the order of the day; and were it not that my course of treatment, in the above case, was pronounced too depletory by veterans in the practice, who have had opportunities of observing ten cases of the kind to my one, I should not have been excited to so much wonder. When, in my defence, I quoted the authority of Dr. Dewees, who, in one case, used these means to a much greater extent, I was reminded that it was not the same disease, or, rather, that it was not under the same circumstances. What Mitchell and Macculloch would have thought of my practice, I am unable to determine; but it is worthy of remark, that the convulsions ceased soon after the blister, which was applied over the superior dorsal vertebræ, began to draw; although there was no tenderness on pressure in any part of the spinal column. Whether the blister had any effect in arresting the convulsions—whether it was the sole cause, or whether it acted in conjunction with the venesection, I am unable to say. I am inclined, however, to believe that it was an important auxiliary to the bleeding. It will be recollected that at 10 o'clock A. M. she lost thirty ounces of blood, which appeared to suspend the convulsions for four hours, but, upon their repetition at 2 P. M., the bleeding had no effect, until the blister began to draw.

